# Novel approaches to meeting the needs of the radiochemistry workforce: a case study of the University of Iowa radiochemistry graduate certificate program

**DOI:** 10.1007/s10967-025-10400-y

**Published:** 2025-10-24

**Authors:** Ecem Celik, Dustin May, Korey P. Carter, Royce Riessen, Sarah Wright, Julianne Nassif, Renee S. Cole, Tori Z. Forbes

**Affiliations:** 1 Department of Chemistry, University of Iowa, Iowa City, IA USA; 2 State Hygienic Laboratory, University of Iowa, Coralville, IA USA; 3 Department of Occupational and Environmental Health, University of Iowa, Iowa City, IA USA; 4 Association of Public Health Laboratories, Bethesda, MD USA

**Keywords:** Radiochemistry, Workforce training, Educational programs

## Abstract

The workforce and training needs for the field of radiochemistry are well documented and continue to be an area of concern for the energy, medicine, environmental monitoring, national security, and forensics sectors. Early efforts to meet these workforce needs have focused on traditional graduate programs (both M.S. and Ph.D.) for training the next generation of radiochemists. However, the changing face of higher education combined with the rigid structure of traditional university programs can lead to development and sustainability challenges for radiochemistry education programs. Some of these issues include a lack of faculty with technical expertise to teach courses, balancing the needs of the department teaching loads, insufficient numbers of students to meet enrollment requirements, and special infrastructure, instrumentation, and safety needed to offer training in radiochemistry. Herein we outline alternative approaches that include virtual/online platforms to enhance current efforts in radiochemistry workforce training, and we highlight our experience developing the University of Iowa’s Radiochemistry Graduate Certificate Program as an example of a new pathway for advancing workforce development that combines both virtual and hands-on instruction. From its launch in Fall 2024 through Summer 2025, the program enrolled 12 participants from different public health laboratories across the United States, with a 100% completion rate. The experience of launching this program has highlighted additional potential growth opportunities to support and develop a comprehensive approach to meeting the national needs for radiochemistry expertise and these are described as well.

**Supplementary Information:**

The online version contains supplementary material available at 10.1007/s10967-025-10400-y.

## Introduction

The field of radiochemistry is critical across a diverse range of areas, yet challenges persist in meeting workforce needs at both the national and global scale. Radiochemists contribute essential expertise in a variety of sectors including nuclear energy, medical diagnostics and therapy, environmental monitoring and remediation, national security, nuclear forensics, and preparedness and response [1–4]. In the nuclear energy sector, radiochemists play a central role in nuclear fuel cycle chemistry, reactor operation, and the management of radioactive waste and materials. Rapid growth in the development of nuclear energy resources is occurring worldwide with a pledge by 22 countries to triple the nuclear power generating capacity on the planet by 2050 [5]. Radiochemists in healthcare support the development and production of radiopharmaceuticals and isotopes for both imaging and treatment, with the global radiopharmaceuticals market valued at approximately $6.45 billion in 2024 and projected to more than double by 2034 [6], reflecting the growing demand for trained professionals in this area. Environmental monitoring and remediation efforts rely on radiochemistry for detecting and managing radionuclide contamination to ensure public health and ecological safety, while national security and forensic applications depend on radiochemists for nuclear material analysis and treaty verification. Despite the increasing importance of these applications, the number of qualified radiochemists remains limited. This gap in workforce readiness has been recognized in multiple national assessments, including reports by the United States Department of Energy (DOE) and the National Academy of Sciences, Engineering, and Medicine, which emphasized the need for expanded education and training pathways in radiochemistry [7, 8]. For example, a 2012 National Academy of Sciences report noted that fewer than fifteen chemistry departments in the United States offer coursework or research opportunities in nuclear or radiochemistry, and only a small number of Ph.D. degrees are awarded annually in the field. While these numbers reflect conditions over a decade ago and may not capture more recent trends, they remain a useful indicator of the scale and persistence of workforce shortages in radiochemistry. Although recent federal initiatives have helped stabilize some of this decline, workforce projections remain concerning since the projected demand for Ph.D. level radiochemists was more than four times the projected supply [9]. This ongoing gap underscores the importance of developing flexible training models that not only prepare new graduate students but also provide opportunities for mid-career professionals to strengthen radiochemistry skills. In the energy sector alone, increasing nuclear capacity to 750 GWe will require about 2.5 million new members of the nuclear workforce worldwide (about 100,000 per year on average) [5]. Furthermore, the combination of an aging workforce, increasing national laboratory and private industry needs, and limited graduate level programs has led to concerns about sustaining expertise in this specialized field. Therefore, these realities underscore the urgency of developing flexible, scalable educational models that can meet current and future workforce demands both in the United States (U.S.) and abroad.

**Fig. 1:**
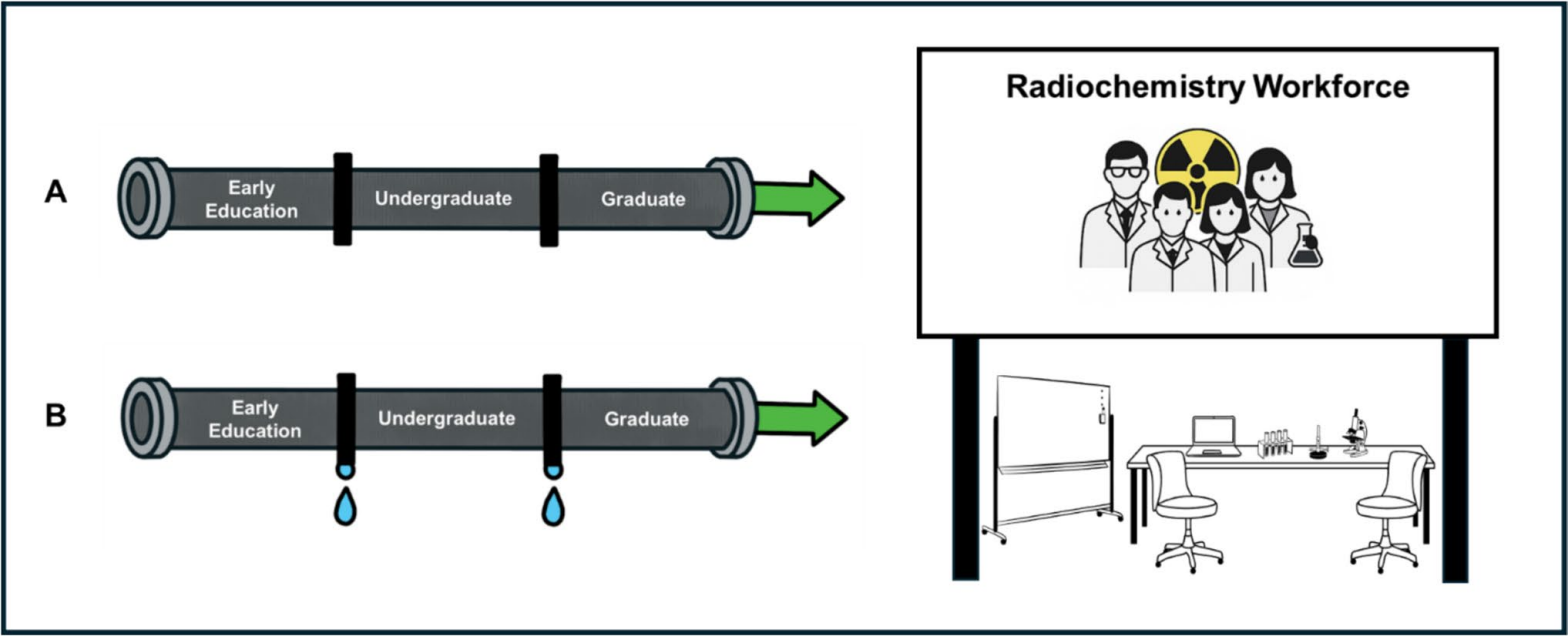
The radiochemistry workforce scheme as (**a**) a traditional pipeline model and (**b**) a leaky pipeline model

Historically, the primary response to radiochemistry workforce demands has been the establishment and maintenance of a workforce pipeline (Fig. 1a). Under this framework, students would be introduced to the idea of radiochemistry at a younger age (i.e., middle or high school) and become part of the training pipeline throughout their undergraduate careers. They would then matriculate into dedicated graduate degrees, such as Master of Science (M.S.) and Doctor of Philosophy (Ph.D.) programs, designed explicitly to produce the next generation of radiochemists. These students would then directly go into the workforce to meet the continued needs of different radiochemistry sectors. While some students do follow this pathway, the low numbers that reach the final employment stage are a result of what has previously been defined as the “leaky pipeline” (Fig. 1b). There are multiple current challenges to the traditional workforce pipeline model including standardized curriculum in primary and secondary education that leaves little time to introduce students to radiochemistry topics [10]. General informal outreach events targeting primary and secondary education have become a common strategy to promote early engagement with Science, Technology, Engineering, and Mathematics (STEM) disciplines, and while they are generally viewed as beneficial, their long-term effectiveness has not been comprehensively explored. Large scale evaluations of general informal outreach events, such as the Science and Engineering Challenge (SEC) in Australia, reported that approximately 30% of student participants indicated the SEC program influenced their decision to pursue STEM education or careers, suggesting measurable, though not universal impact [11]. However, other studies highlight that while outreach programs may enhance interest and perceptions of science in students, these changes do not consistently result in sustained engagement or progression toward STEM careers, particularly in specialized fields such as radiochemistry [12, 13]. In response to these challenges, some efforts have been devoted towards the development of case studies that allow for embedding nuclear concepts within classroom settings through scalable and curriculum aligned tools. For instance, Lu et al*.* developed a gamified card activity, RAD Ratings, designed to teach nuclear science concepts to secondary education students [14]. The intervention demonstrated improved conceptual understanding and classroom engagement when implemented in structured lessons. However, while these outcomes indicate short-term gains in STEM interest and comprehension, the study did not assess whether such interventions influence students’ long-term educational or career pathways in science. Overall, these findings reinforce the need for continuous and scaffolded exposure to science and structured educational pathways that extend beyond single-point interventions.

Once students enter college in the U.S., there are no dedicated undergraduate programs except a small number of programs offering a B.S. in chemistry with a concentration in radiochemistry [15]. There are some high quality experiential programs at the undergraduate level that offer a strong pipeline into graduate degrees, with the DOE/American Chemical Society (ACS) Nuclear Chemistry and Radiochemistry Summer School a hallmark example. However, these programs must compete with other summer research experiences, and the hands-on nature of the program necessitates small enrollments. Graduate programs in radiochemistry provide solid foundational training, but they often face significant limitations in adaptability and sustainability, especially for those who are already in the workforce, impacting their effectiveness in addressing evolving workforce needs. Compounding these problems, traditional undergraduate and graduate programs in radiochemistry typically require significant institutional resources, specialized infrastructure, laboratory spaces, instrumentation, and rigorous adherence to strict safety regulations. Additionally, these programs are highly dependent on having faculty members with specific technical expertise in radiochemistry, which can be a limiting factor due to retirements, shifts in research focuses, and budgetary constraints [16]. Consequently, departments frequently struggle with maintaining consistent course offerings in radiochemistry, worsened by small student enrollments that challenge minimum requirements for sustained program funding and administrative support.

On top of this, the higher education landscape itself has been undergoing considerable transformation, placing additional pressures on traditional educational models. Budgetary reductions, shifts toward interdisciplinary and applied fields, emphasis on shortened times to degrees, and the need to balance teaching and research responsibilities further strain the abilities of the departments to consistently support specialized training programs. As a result, the traditional educational pipeline metaphor, in which students progress linearly from high school through undergraduate to graduate education in a specialized field, such as radiochemistry, until they graduate and use that education in the workforce, is increasingly vulnerable to disruptions and limited in its ability to respond to real-time workforce demands [17].

**Fig. 2:**
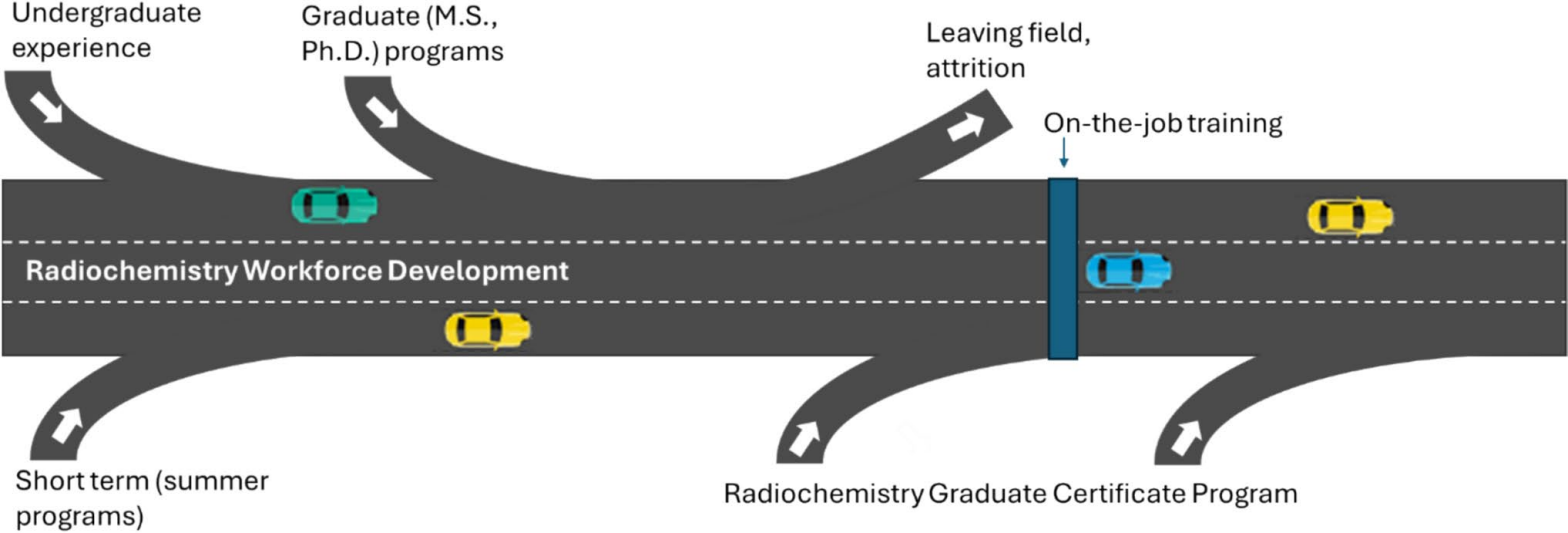
The Radiochemistry Workforce Development highway model has multiple entry and exit ramps where students can enter or exit the field. The University of Iowa Radiochemistry Graduate Certificate program acts as an “on-ramp” helping individuals enter the radiochemistry workforce either during graduate school or after they have secured a position in the field. In the figure, this role is represented by the on-ramps on either side of the blue on-the-job training section, which highlights the certificate program’s position as a structured pathway into the workforce

Moreover, the traditional pipeline analogy does not account for the many decision points that students face as they try to navigate their way to during a career, missing opportunities to address immediate radiochemistry workforce gaps in critical sectors. A better model is a career highway that has multiple points for students to choose an off-ramp from or on-ramp into the workforce (Fig. 2). There are multiple places within degree pathways wherein individuals often exit undergraduate or graduate programs without specific radiochemistry expertise, leading to off-ramps that contribute to difficulties in addressing immediate radiochemistry workforce gaps in critical sectors. However, there are many individuals who might contribute effectively to radiochemistry positions because basic laboratory skills learned at the undergraduate level can be transferable with additional training. Similarly, most graduate students originate from the traditional subdisciplines of chemistry (organic, physical, analytical, or inorganic chemistry) and develop skills, including separations techniques, spectroscopy, and instrumental material characterization, that are highly transferable to radiochemical contexts. There remains a critical need though to further develop these later career on-ramps that can enable students to add specific radiochemistry skills to the generalized chemistry knowledge obtained through traditional degrees.

Recognizing the urgent need for flexibility and responsiveness to dynamic workforce conditions amid a shortage of trained radiochemists, we set out to develop an alternative non-traditional educational mechanism that offers continuing education opportunities for those currently in the workforce. Our efforts to create a radiochemistry program at the University of Iowa (UI) was planted as a seed in 2012 and have substantially grown to date. In this perspective, we specifically highlight our experiences developing the Radiochemistry Graduate Certificate Program, which strategically integrates asynchronous modular online learning through virtual platforms (e.g., Canvas course management) with focused content and intensive hands-on practical experiences. This novel approach offers flexible learning environments with interactive components to foster an engaging learning experience, allowing working professionals in related areas to gain specialized radiochemistry knowledge, thereby transforming them into highly competent radiochemists. By discussing the unique advantages and growth potential of this graduate certificate model, we aim to illustrate an alternative, robust, and adaptable approach to workforce training. Grounded firmly both in cognitive and educational science research, this comprehensive, long-term modular educational program significantly advances professional radiochemistry education, ensuring deeper learning, enhanced retention, and more effective application of skills within professional environments. Overall, this program not only addresses immediate training needs but also supports long-term growth opportunities in the workforce, making a significant contribution to national and international efforts focused on sustaining expertise in radiochemistry.

## Current ideas: comparative approaches in continuing radiochemistry education

Employees across industries are generally expected to increase their skills and knowledge to contribute to the mission of their company or institution through either structured (courses, workshops, on-the-job training) or unstructured (independent readings, scientific talks, professional communications, shadowing) professional development opportunities. In this section, we will focus on structured forms that provide potentially greater opportunities for people to join the radiochemistry workforce pipeline. Within the field, numerous structured programs have been established to support education and workforce development over many decades. Formal academic pathways include graduate coursework offered at institutions such as University of California, Berkeley, Washington State University, the University of Nevada, Las Vegas, Clemson University, University of California, Irvine, Oregon State University, Florida State University, Colorado School of Mines, and Colorado State University, each providing advanced courses in radiochemistry, actinide chemistry, or environmental nuclear science. A summary of universities listed on the American Chemical Society Nuclear Chemistry and Technology Division with graduate programs related to nuclear chemistry and technology and their offerings is provided in Section S1, Supporting Information. Beyond traditional academic settings, several well-established workshops and short-term programs offer intensive training as well. These include the DOE/ACS Nuclear Chemistry and Radiochemistry Summer School, a six-week hands-on program for undergraduates, the Nuclear Forensics Summer School hosted at national laboratories sponsored by the DOE, the Undergraduate Radiochemistry Summer School at Oregon State University, and the DOE’s National Analytical Management Program (NAMP), which provides specialized training in radiochemical methods such as alpha spectroscopy. Additional offerings include the U.S. Environmental Protection Agency (EPA) Radiochemistry Training Series and professional development workshops hosted by the American Nuclear Society (ANS). These programs collectively demonstrate the breadth of professional learning opportunities currently available to support entry into and advancement within the radiochemistry workforce. We recognize that there are strengths and weaknesses to each type of program and have summarized our takeaways in Table 1.

**Table 1:** Comparison of Structured Professional Development Settings for Enhancing Radiochemistry Workforce Training

Setting	Strengths	Weaknesses
On-the-Job Training	∙ Context-specific and directly applicable ∙ Builds familiarity with site-specific procedures and equipment ∙ Low external cost ∙ Embedded within work schedules ∙ No need for extra commute or time away from work	∙ Highly variable quality ∙ Often lacks theoretical foundation-training on the how not the why ∙ Skills learned-knowledge may be very specific to the organizations ∙ No formal academic recognition ∙ Learning environments can be high-pressure
Workshops and Seminars	∙ Useful for raising awareness or initial exposure to a topic ∙ Can spark interest and motivation for further learning ∙ Appropriate for targeted, low-complexity skill acquisition ∙ Often aligned with specific professional needs or niche topics ∙ Low time commitment ∙ Cost-effective and widely accessible	∙ Limited depth of content and knowledge ∙ Surface level engagement, prioritizing content exposure over conceptual understanding ∙ Minimal follow-up, feedback, or assessments included ∙ No hands-on learning experience ∙ May not be transferable across domains or organizations ∙ No formal academic credentials
Short-Term Programs	∙ Good for introductory exposure and topic awareness ∙ Can offer immersive, structured learning in a short period ∙ May include limited hands-on activities if lab infrastructure is available ∙ Can act as an entry on-ramp into radiochemistry for early-stage learners ∙ Low time commitment ∙ Somewhat cost-effective depending on sponsorship or location ∙ May provide opportunities for networking, community-building	∙ Limited content depth or time for conceptual engagement ∙ May have minimal follow-up or assessments included ∙ Hands-on components, if present, may be short or constrained by logistics ∙ No formal academic credentials, but may earn college credits ∙ May be limited capacity (e.g., 20 participants/year in federally funded programs) ∙ Competitive admission processes may restrict access
Formal Graduate Coursework	∙ Scaffolded instruction with learning outcomes ∙ Emphasis on both theory and application ∙ Includes structured assessments and feedback mechanisms ∙ Supports deeper levels of cognitive engagement ∙ May include research or lab-based components depending on course design ∙ Formal academic credential with transferable credits for further education	∙ Higher time and financial commitment ∙ Requires enrollment in a university system with its associated prerequisites ∙ Dependent on institutional infrastructure and departmental offerings ∙ Incompatible course scheduling with work schedules ∙ Requires physical attendance or synchronous participation, limiting flexibility ∙ May not focus on workforce-specific skills unless designed for applied contexts ∙ Deadline for completion
Graduate Certificate Program	∙ Offers structured pathway without requiring full graduate degree enrollment ∙ Formal academic credential recognized by institutions and employers ∙ Transferable credit for further education ∙ Scaffolded, modular content ∙ Emphasis on both theoretical knowledge and applied problem-solving ∙ Supports higher-order cognitive outcomes ∙ Compatible with work schedules ∙ Allows for flexible pacing ∙ Regular assessments and feedback mechanisms ∙ Encourages long-term engagement through milestone-based progress	∙ Higher time and financial commitment ∙ Require some university infrastructure and resources ∙ Still requires institutional admission even if not into a full degree program ∙ May involve time and travel for practical hands-on laboratory courses

To systematically build expertise in a complex topic such as radiochemistry, educational interventions should progress from fundamental exposure to advanced cognitive engagement [18]. Applying Marzano’s taxonomy as a guiding framework, we highlight the pedagogical considerations that led us to develop a graduate certificate program addressing current needs and unmet gaps in radiochemistry training. Marzano’s taxonomy categorizes cognitive processes into four hierarchical levels: retrieval, comprehension, analysis, and knowledge utilization, each reflecting progressively deeper cognitive skills [19]. Employing this taxonomy allows us to clearly align educational interventions ranging from on-the-job training (OJT) to a comprehensive graduate certificate program with their targeted outcomes. Through this perspective, we summarize both the strengths and limitations of current instructional approaches that are listed in Table 1, emphasizing the need for structured, modular graduate certificate programs to cultivate deeper knowledge comprehension, utilization, and transferable expertise in radiochemistry.

### On-the-job training (OJT)

On-the-job training (OJT) represents a common approach to professional education, particularly within specialized technical fields such as radiochemistry. A major benefit of OJT is that organizations can build context specific skills that are tailored to site-specific procedures and equipment. Training takes place within the work schedule, which enables employees to maintain work-life balance and does not require additional travel to external sites. While offering practical, context-specific, and job-related experiences, OJT does have some weaknesses. One limitation is a lack of theoretical foundation since OJT trainings are typically focused on how a process works rather than explaining the fundamentals. These informal training environments also typically lack structured objectives, consistent quality, formal evaluation methods, and feedback mechanisms that are essential elements identified by contemporary educational research as crucial for effective learning outcomes [20]. For instance, the absence of structured reflection and intentional practice within a workplace learning environment can result in fragmented or superficial understanding, limiting effective knowledge transfer to new or varied situations. To most effectively learn new information, adults working in complex cognitive environments benefit from structured, step-by-step instruction, which contrasts with the informal and often unstructured nature of many OJT trainings [21, 22]. In these less formal settings, professionals are typically expected to learn by doing or by asking for help when needed, with minimal guided support. In some cases, the high-pressure nature of the workplace can further discourage experimentation, questioning, or reflection, which are key components of effective adult learning [23].

The effectiveness of workplace training is often evaluated using a four level framework that consists of learners’ reactions, knowledge acquisition, transfer of learning to the job, and return on investment for the organization [23]. While the first two levels are frequently assessed, the third component is both more difficult to evaluate and arguably the most important for meaningful professional development. Therefore, the transfer of learning is more likely to occur when training incorporates realistic difficulties, reflects the actual work context, and is supported by peers and supervisors post-training. In many cases, OJT lacks these structured supports, making successful transfer less reliable and more variable across individuals and settings [24]. Moreover, OJT environments are rarely individually tailored to account for learner characteristics, such as age, prior knowledge, or motivation. Studies show that adult learners, for example, benefit from structured and scaffolded instruction, yet these accommodations are typically absent in informal settings [23]. Tailored instruction enabled through different nontraditional technological settings allows learners to dynamically adjust instructional content and pacing, which has been shown to improve outcomes, especially in cognitively complex environments [25]. This adaptability is particularly valuable in radiochemistry, where foundational understanding must be solid before progressing to applications involving safety and regulatory compliance. Additionally, OJT does not result in formal academic credentials, limiting its recognition and value in broader professional or academic advancement pathways.

### Workshops and seminars

Continuing education in specialized disciplines, including radiochemistry, traditionally relies heavily on graduate programs as well as workshops and seminars. Among these, technical workshops and seminars are particularly attractive due to their minimal time commitments, flexibility, and targeted content delivery, which is highly compatible with professionals seeking rapid skill enhancement or introductory exposure to a certain topic. These offerings can take place online, which provides broad reach and accessibility. Moreover, workshops and seminars are often offered at scientific conferences, and this provides additional opportunities for professional networking. Despite these practical benefits, the briefness of such programs significantly limits the depth and durability of content that can be learned. While workshops can be effective solutions for learning a specific experimental technique or software, they are not suited for delivering large volumes of information or cultivating deep understanding that can be applied across varied professional contexts. A misalignment between intended outcomes and the nature of professional development often leads to a quick gain in awareness of a topic but only surface-level knowledge, resulting in superficial understanding, limited engagement, and inadequate comprehension of complex concepts such as radiochemistry.

Cognitive science research underscores that deep and lasting knowledge acquisition necessitates repeated exposure to concepts, active cognitive engagement, and sufficient time intervals between learning sessions to consolidate information effectively and enable active recall [23, 26]. Memory research also emphasizes the advantages of distributed practice, indicating that knowledge retention and memory consolidation significantly improves when learning sessions are spaced over extended periods rather than condensed into short time intervals [27–29]. This repeated exposure and practice not only supports better long-term memory retention but also promotes deeper conceptual understanding, comprehension, and more effective utilization of learned skills in professional contexts. Workshops and seminars rarely provide the time or structure necessary for critical reflection, repeated practice, or the cognitive integration of complex topics. Brief educational interventions infrequently allow for continuous learning cycles such as practicing, reflecting, receiving constructive feedback, and adapting, which are essential components for meaningful learning [23]. Additionally, workshops and seminars often induce cognitive overload due to their compressed schedules, limiting the capacity of participants to absorb and comprehend new information [30]. This overload can often lead to only partial processing of content, making it challenging for individual learners to develop the deep conceptual networks required for effective transfer and application in professional workforce settings. Workshops and seminars also frequently prioritize content breadth over depth, resulting in surface-level engagement with complex material, and developing meaningful understanding requires more than passive transmission of facts, it depends on well-organized knowledge frameworks and opportunities to reflect, apply, and integrate new material into existing schemas [31]. Effective learning involves conceptual restructuring, transfer, and metacognitive awareness, all of which are rarely addressed in these short-format sessions [32]. These characteristics limit the ability of workshops and seminars to support knowledge utilization, or professional adaptability, particularly in fields like radiochemistry that demand both theoretical grounding and practical application.

### Short-term programs

Short-term programs, such as summer schools or intensive multi-day training courses, offer condensed educational experiences and can be designed to introduce participants to specialized topics like radiochemistry. These programs are especially attractive for students or early-career professionals seeking initial exposure to the field without committing to a full academic semester or degree program. Their structure typically lasts from a few days to multiple weeks, which makes them appealing for their logistical convenience, lower cost, and ability to accommodate a wide range of participants from different institutions or backgrounds. When well-designed, such programs can serve as effective early on-ramps into the field, offering immersive exposure that sparks interest, builds foundational terminology, and encourages further exploration. In some cases, they may also provide opportunities for networking, community-building, or initial hands-on experience, particularly in federally supported summer programs.

Despite their accessibility and utility for introductory exposure, short-term programs face notable constraints in delivering content depth and enabling material comprehension. While some programs incorporate minimal hands-on experiences, such opportunities are often limited by time, resources, and limits in the number of participants. In many cases, content must be condensed while leaving little room for repetition, reflection, or extended application exercises that support lasting conceptual understanding. Short-term programs may also introduce some of the key ideas or experimental techniques relevant to a topic but rarely allow learners to progress beyond surface-level familiarity. Additionally, their short duration limits opportunities for learners to receive feedback, engage deeply, or revisit materials, all of which are known to support cognitive consolidation and transfer of knowledge into utilization [23, 26]. Capacity is another major limitation in these kinds of programs that are usually funded through national agencies or hosted by specialized institutions, which often serve only a small number of participants annually, limiting broader workforce impact. Further, while short-term programs may raise interest in radiochemistry and support awareness-building, they typically do not confer formal academic credentials or structured milestones that support professional advancement. These limitations are particularly relevant in technical fields like radiochemistry because without continuous engagement with complex content, learners may struggle to integrate new concepts into their existing knowledge structures or apply them effectively in real-world scenarios [33].

### Formal graduate coursework

Graduate-level coursework remains one of the most established pathways for advancing technical knowledge in specialized disciplines such as radiochemistry. These courses typically provide scaffolded instruction, formal academic credit, and clearly defined learning outcomes, making them a valuable option for learners seeking both theoretical grounding and applied skill development. Instruction is often delivered on-campus by subject matter experts, with curricula designed to support cognitive engagement through lectures, assessments, discussions, and, occasionally, lab-based activities. Therefore, well-designed coursework that incorporates effective learning principles has the ability to promote comprehension and analysis by guiding learners through progressively complex material and offering structured opportunities for reflection and evaluation [34].

In addition to its academic rigor, coursework also confers transferable academic credentials, which can support advanced degree completion or further graduate study. These features make traditional coursework an important contributor to radiochemistry education, particularly for students enrolled in graduate degree programs or those with flexible schedules. However, access to graduate courses often requires formal admission to a university or department. In some cases, coursework is accessible to non-degree-seeking students, but institutions often limit the number of credits such students may take, and tuition costs can be prohibitively high. Scheduling constraints, such as fixed meeting times and synchronous delivery, may also present additional challenges for working adults balancing multiple responsibilities. While coursework supports deep understanding, it may also not be fully optimized for workforce readiness unless designed with applied, professional contexts in mind. In fields like radiochemistry, where theory must be paired with real-world applications, the extent to which coursework supports knowledge utilization depends heavily on the course structure and learning environment [35].

### Graduate certificate program

Graduate certificate programs represent a structured and academically rigorous approach to workforce development that bridges the gap between informal training and full graduate degrees for working professionals. In fields such as radiochemistry, where both conceptual understanding and application competence are critical, this format offers an accessible on-ramp and high-impact solution especially for people who are already in the workforce seeking formal credentials without committing to a multi-year graduate program. The modular, scaffolded design of graduate certificate curriculum enables learners to engage with foundational material before progressing to more advanced topics, supporting comprehension and knowledge utilization over time.

One of the key advantages of graduate certificates, and our model in particular, are their flexibility. While programs can be completed in 1–2 years with a standard course load, there are no fixed deadlines for completion allowing learners to take fewer courses in a given term and/or extend the timeline if needed to accommodate their work schedules, caregiving responsibilities, personal preferences, or other life circumstances. This characteristic makes certificate programs particularly compatible with the needs of working professionals, allowing them to pursue professional development without stepping away from their current roles and responsibilities. Similar to other graduate-level programs, our certificate program also requires university infrastructure and resources; however, asynchronous lecture content delivery and milestone-based progression provide additional flexibility to take the courses wherever and whenever is best for students. Academically, graduate certificates confer a formal credential that is recognized by both academic institutions and employers. Learning outcomes are reinforced through structured assessments, regular feedback, and real-world scenario-based problem-solving activities that align with the higher levels of Marzano’s taxonomy, including analysis and knowledge utilization [19, 36]. These features encourage learners not only to understand and comprehend radiochemistry concepts but also to apply them in novel contexts, solve problems, and make informed decisions within their real-world professional settings. While this ensures academic rigor and quality, it also entails a higher time and financial commitment than other informal learning (e.g., workshops and seminars) opportunities. However, for those seeking a coherent, comprehensive, and flexible academic pathway within the radiochemistry workforce, a graduate certificate program offers a strategic entry point to an accessible on-ramp that enables both foundational training and advanced skills development.

## The development, current situation and growth of radiochemistry graduate certificate program at the University of Iowa

The Radiochemistry Graduate Certificate Program at UI is the first in the nation that offers a continuing education opportunity for those currently in the workforce. In response to the national shortage of trained radiochemistry professionals, we developed a graduate certificate program tailored specifically to people holding Bachelor of Science (BS) or Bachelor of Arts (BA) degrees who are already embedded in the workforce. Unlike traditional graduate degree programs, which typically demand substantial time, financial resources, and commitments that are often impractical for working professionals, our graduate certificate model offers flexibility and is based on pedagogical best practices to maximize effective learning, enabling participants to gain specialized radiochemistry expertise within 1 year. Our program spans three semesters that are fall, spring, and summer, totaling 12 semester hours. A cumulative grade point average (GPA) of 2.5 or higher is required for acceptance into the certificate program, and maintaining this same grade point average is the requirement for completing the program. The yearlong modular format maintains academic rigor while increasing accessibility and providing more flexibility compared to traditional settings, allowing professionals to gain credentials that can enhance their qualifications without disrupting their careers. A one-page program at a glance is further provided in Section S2, Supporting Information.

Our certificate program was developed through a collaborative partnership between the University of Iowa and the Association of Public Health Laboratories (APHL), who provided key insights into the skill gaps and workforce needs within public health laboratories across the country, initial financial support for program development, and student scholarships. The involvement of APHL ensured that our modular course content would be aligned with real-world responsibilities as well as safety expectations in radiochemical monitoring and analysis, especially in the context of public health. We created this program to directly address APHL’s current strategic workforce development goals and to serve as a national model for aligning academic training with public health needs. Our current course offerings are described in detail in Section S3, Supporting Information.

The other key component to the success of our certificate program is the robust support provided by the University of Iowa’s Office of Distance and Online Education. These administrative partners have been essential in developing technical infrastructure and ensuring that we could create professional, engaging content for asynchronous online lectures via tools such as a recording studio featuring a green screen, the ability to provide and record on-screen annotations, and a lightboard. All content creation was done in concert with Distance and Online Education staff with professional expertise in lighting and sound editing that enabled course lectures to be delivered asynchronously with high production quality, in alignment with the needs of public health radiochemistry working professionals across the country.

**Fig. 3:**
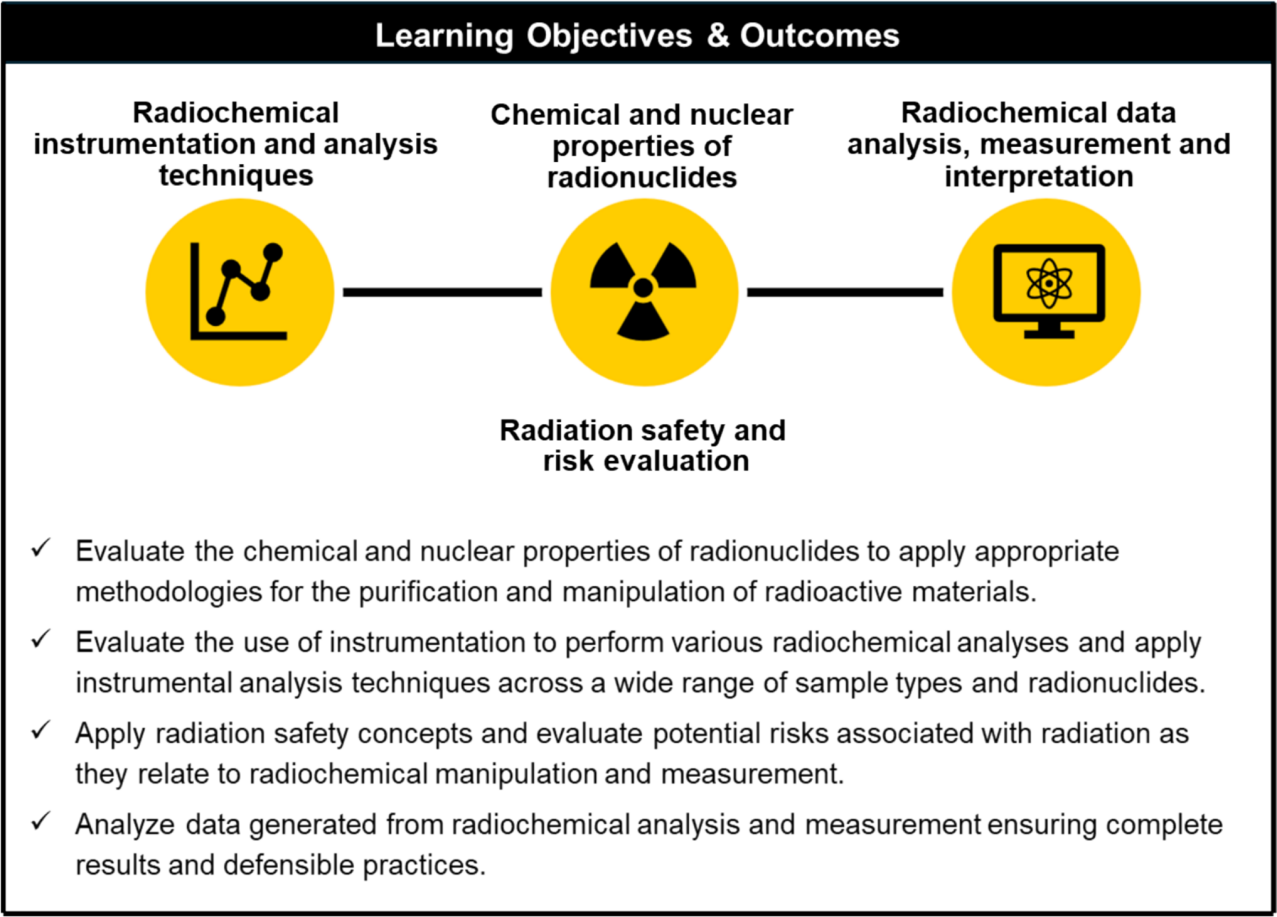
Program learning objectives and outcomes for The University of Iowa Radiochemistry Graduate Certificate Program

At the foundation of this program is a carefully structured set of learning objectives and measurable outcomes (Fig. 3). These were developed in collaboration with the radiochemistry faculty members and the UI Distance Learning Team to ensure that the certificate not only provides foundational knowledge in radiochemistry but also prepares learners to apply what they have learned directly in laboratory and field environments. Our learning outcomes emphasize both fundamental and conceptual understanding and cover a range of topics including nuclear physics concepts, radiochemical separations, radiation safety and health physics, data analysis and statistics, regulatory compliance and quality assurance practices, and radiochemical instrumental analysis. Upon completion, our certificate program equips professionals with expertise in radiochemical analysis, measurement techniques and characterization, radiation safety and risk mitigation, and data analysis and interpretation. This is accomplished through completion of asynchronous content modules and in-lab applied practice with a focus on Environmental Protection Agency (EPA) and American Society for Testing and Materials (ASTM) methods (i.e., 900.0, 903.1, 904.0, 906.0 etc.). More specifically, students who complete the program will be able to evaluate the chemical and nuclear properties of radionuclides, apply appropriate methodologies for the purification and manipulation of radioactive materials, use instrumentation to perform various radiochemical analyses, apply instrumental analysis techniques across a wide range of sample types and radionuclides, apply radiation safety concepts to evaluate potential risks associated with radiation as they relate to radiochemical manipulation and measurement, and analyze data generated from radiochemical analysis and measurement to ensure complete results and defensible practices.

**Fig. 4:**
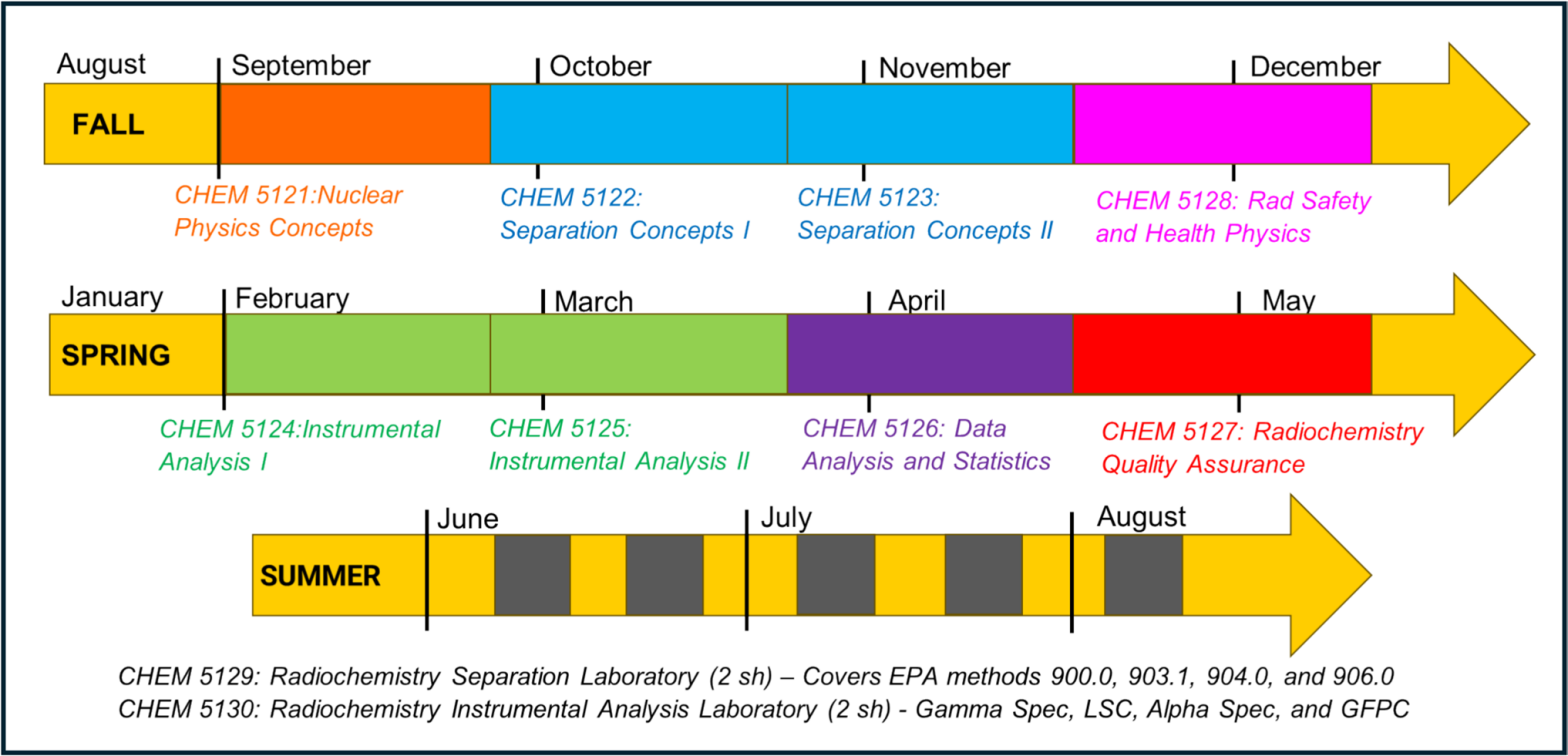
The curriculum outline of the Radiochemistry Graduate Certificate Program at the University of Iowa. Courses are color-coded by outcome domain (nuclear physics = orange, separation = blue, safety = pink, instrumental analysis = green, data analysis/statistics = purple, quality assurance = red). (Color figure online)

The curriculum was designed using a modular approach in which each one-semester-hour course targets a specific topic within the broader discipline of radiochemistry (Fig. 4). We offer flexible learning environments with interactive content to foster an engaging student learning experience. Each module includes professionally recorded asynchronous lectures, tailored course materials including readings and assessments (i.e., quizzes, assignments, and final exams) clearly organized within the Canvas course management platform, and virtual live weekly Zoom office hours held by the course professor. Teaching assistants also provide additional support through office hours by appointment, feedback on assignments and exams returned within 2–3 days, and responses to questions via e-mail typically within 24 hours. Students complete one to three modules per week, requiring approximately three hours of engagement per module. Each module includes short, pre-recorded lecture videos (10–15 min each), which are intentionally kept brief to maximize engagement and align with evidence that shorter segments improve focus and retention in online learning environments. In addition, each module incorporates a short multiple-choice quiz (five to six questions) based on the video content and there are weekly problem sets (two to three questions) to reinforce concepts. Upon completion of the asynchronous portion of the certificate program, students complete a two-week intensive hands-on lab experience led at the State Hygienic Laboratory (SHL) at UI. To ensure consistent evaluation, we have implemented a practical skills rubric that provides a structured and transparent framework for assessing student performance during the laboratory courses (Section S4, Supporting Information). Our program also incorporates continuous feedback mechanisms to ensure ongoing relevance and to provide pathways for improvement of our structure and curriculum. Feedback from student evaluations, APHL partners, and external reviewers such as our dedicated teaching assistants is used to revise course materials annually. Moreover, our professors remain actively engaged in the field and adapt course content to reflect changes in best practices. Creating a sustainable program is a key focus of our current efforts and future course modules under development will address workforce needs in sectors such as nuclear medicine, nuclear energy, and nuclear forensics with the goal of creating a dynamic, scalable educational platform that grows and adapts as radiochemistry evolves. This modular and flexible structure allows participants to customize their learning pathway based on professional background, institutional needs, or emerging national priorities in radiochemistry. The preliminary program outcomes for the first cohort are reported in Section S5 Supporting Information.

## Increasing the flow: current challenges and opportunities to expand

### Expansion of current offerings

The current curriculum was originally designed in close partnership with the APHL to address critical workforce gaps in public health laboratories. The modular nature of the short, focused, one-semester-hour courses has proven especially effective for professionals already in the workforce. Moreover, this flexibility creates a foundation for the future expansion of our current offerings. We can continue to add one semester-hour courses and new modules that can be strategically developed to support sector-specific applications, such as energy, medicine, and national security. This targeted expansion would allow professionals who are in the workforce across these sectors to gain access to core radiochemistry knowledge and technical competencies needed to advance within their roles, while maintaining the program’s adaptable format for adult learners who are already in the workforce. Additional university funding would enhance the expansion of the program to include students beyond the public health laboratories and enable focus in other radiochemistry specialties, such as nuclear energy and medicine, to ensure sustainability of the program. Over the next 12–18 months, we plan to develop new modules in nuclear medicine and energy, with a target cohort size of ~ 25–30 learners. Looking further ahead, additional modules are planned for development in 2026–2027, with the goal of offering an expanded curriculum beginning in Fall 2027. In addition, a professional master’s program building on the certificate is also being scoped with internal review planned for 2026–2027 and a planned launch in Fall 2027.

### Measuring learning and long-term impacts

The current evaluation framework for the certificate program includes both formative and summative measures, such as feedback provided through graded problem sets, and examinations for the modular lecture-based courses, as well as a practical skills rubric to assess student performance during the laboratory component. Future cohorts will incorporate additional measures to strengthen program assessment. Pre- and post-content assessments, aligned with the stated learning outcomes, will provide a direct measure of student learning gains across the online modules. In addition, a 3–6 month follow-up survey will be developed both to document how participants apply knowledge and skills in their workplace and to ensure that the program continues to meet both student and workforce needs. These additions will extend evaluation beyond participation metrics, offering a clearer picture of student outcomes and helping guide continuous improvement of the program.

### Establishing links to different sectors

With the development of courses for different areas within radiochemistry, we need to ensure that we are meeting the real-world needs of the workforce. This means that the new curriculum should be vetted by professionals in the field to ensure that content is timely and relevant, and it is essential to establish formal linkages with professionals in the relevant sectors. In addition, engaging with experts in different areas of radiochemistry and regularly reviewing the ongoing curriculum will help validate content and maintain alignment with emerging technologies and field expectations. Having routine feedback cycles such as regular course evaluation and revision grounded in both learner feedback and industry input in the instructional design process can improve course quality and maintain its relevance. Furthermore, post-certificate outcomes (e.g., role changes and workplace impacts) data will be collected for future program assessments. By involving professionals in these efforts across government, industry, and research in different sectors, our certificate program can continue to serve as a bridge between academic preparation and practical application.

### Understanding the costs are worth it

Formal academic programs, including certificates, will have a price tag. This is because universities have to recoup the costs of faculty time, student support, and administration costs related to program operation and degree conferral. The multitude of benefits from program participation include a more rigorous and scientifically grounded understanding of the principles underlying participant’s daily work, which can pay dividends via improved process management, enhanced troubleshooting abilities, greater understanding of how to ensure regulatory compliance, improved laboratory quality assurance practices, and increased potential for job advancement. As employers seek candidates who combine practical skillsets with strong theoretical grounding, completion of our certificate can serve as a differentiator in a competitive workforce enabling graduates to address knowledge gaps within the field and providing justification for the initial financial investment. All details including costs and logistical considerations involved in our program implementation are included in Section S6, Supporting Information.

### Collaborations instead of competition

As educational institutions across the nation recognize the workforce gap and needs, there may be growing interest in launching radiochemistry focused certificate programs. However, having many programs try to start their own certificate programs will lead to challenges related to low enrollments described above. Rather than competing for limited resources, there are areas of collaboration that could strengthen individual institutions and create a national network that collectively supports workforce needs. One area for possible collaboration is hands-on laboratories. While we do have some capacity at the University of Iowa to host in-person hands-on training, expanding these opportunities through regional partnerships could reduce travel costs and improve accessibility by offering on-site or closer to home experiences for working professionals in the program. Our current laboratory capacity at the SHL is constrained by space, equipment, and staffing, which limits the number of participants that can be accommodated each summer (Section S7, Supporting Information). In addition, there are other closely related fields that have similar workforce challenges (i.e., health physics, nuclear engineering, radiation sciences), and cross-disciplinary collaborations with other partner institutions could establish a nationwide radiochemistry network to build up a cohesive and scalable network to help realize the goal of creating a long-term sustainable workforce development pipeline. Reliance on institutional infrastructure further highlights the importance of collaboration, as no single site can independently meet the scale of national workforce demand. These limitations point to the value of developing a ‘regional hub model’ (Section S7, Supporting Information) where shared facilities and expertise can collectively expand training capacity and ensure broader accessibility.

## Conclusions

The need for trained radiochemists to support current and future workforce development is well documented, and addressing this need requires creative strategies to expand the number of “on-ramps” into the field. In this perspective, we evaluated the strengths and weaknesses of existing approaches—including on-the-job training, workshops and seminars, short courses, and formal graduate curricula—and highlighted the University of Iowa Radiochemistry Graduate Certificate Program as a novel model for workforce development. Challenges remain in scaling the certificate program to meet national and international training needs, particularly in terms of cost and the expansion of hands-on training and coursework, and future collaborations and the development of a coordinated national training strategy could significantly strengthen efforts in this area.

## Supplementary Information

Below is the link to the electronic supplementary material.

Supplementary file1 (DOCX 52 KB)
