# Supplementary material for: Novel approaches to meeting the needs of the radiochemistry workforce: a case study of the University of Iowa radiochemistry graduate certificate program
Source: J Radioanal Nucl Chem. 2025 Oct 24;334(12):9195–207. doi: 10.1007/s10967-025-10400-y (PMC12827311; doi:10.1007/s10967-025-10400-y)
Supplement: Supplementary file 1 — Supplementary file1 (DOCX 52 KB) [file 10967_2025_10400_MOESM1_ESM.docx]

Supporting Information Section

***Novel Approaches to Meeting the Needs of the Radiochemistry Workforce: A Case Study of the University of Iowa Radiochemistry Graduate Certificate Program***

Ecem Celik,^a^ Dustin May,^b, c^ Korey P. Carter, ^a^ Royce Riessen,^d^ Sarah Wright,^d^ Julianne Nassif, ^d^ Renee S. Cole,^a^ and Tori Z. Forbes^a*^

*^a^ Department of Chemistry, University of Iowa, Iowa City, IA; ^b^ University of Iowa State Hygienic Laboratory, Coralville, IA; ^c^ Department of Occupational and Environmental Health, University of Iowa, Iowa City, IA; ^d^Association of Public Health Laboratories, Bethesda, MD*

** Corresponding Author,* [*tori-forbes@uiowa.edu*](mailto:tori-forbes@uiowa.edu)

**Table of Contents**

[**S1. U.S. RADIOCHEMISTRY RESEARCH AND PROGRAMS** 1](#_Toc207878792)

[**S2. PROGRAM AT A GLANCE – UNIVERSITY OF IOWA RADIOCHEMISTRY GRADUATE CERTIFICATE** 5](#_Toc207878793)

[**S3. COURSE DESCRIPTIONS** 7](#_Toc207878794)

[**S4. PRACTICAL SKILLS RUBRIC FOR LABORATORY COMPONENTS** 9](#_Toc207878795)

[**S5. PRELIMINARY PROGRAM OUTCOMES** 11](#_Toc207878796)

[**S6. COSTS AND LOGISTICS** 12](#_Toc207878797)

[**S7. STATE HYGIENIC LABORATORY (SHL) CONSTRAINTS AND REGIONAL HUB MODEL CONSIDERATIONS** 13](#_Toc207878798)

# **S1. U.S. RADIOCHEMISTRY RESEARCH AND PROGRAMS**

**Table S1.** Universities that are listed on the American Chemical Society Nuclear Chemistry and Technology Division (www.nucl-acs.org) with graduate programs related to nuclear chemistry and technology. The home department is also listed with the total courses offered in radiochemistry (based upon search of online course catalogues) also provided.

| **University** | **Department** | **Formal Degrees** | **Courses offered in Radiochemistry** |
| --- | --- | --- | --- |
| Colorado School of Mines | Chemistry | Nuclear Engineering | CHGN411 – Applied Radiochemistry |
| Colorado State University | Environmental and Radiological Health Sciences | Radiological Health Sciences | ERHS 665 – Radiochemistry |
| Clemson University | Environmental Engineering |  | EES 6100 – Environmental Radiation Protection I  EES 6110 – Ionizing Radiation Detection and Measurement  EES 6111 – Ionizing Radiation Detection and Measurement Laboratory  EES 6120 – Nuclear Fuel Cycle and Radioactive Waste Management  EES 6140 – Radioecology  EES 8130 – Environmental Radiation Protection Laboratory  EES 8131 – Environmental Radiation Protection Laboratory |
| Florida International University | Chemistry |  | CHS 4100 – Radiochemistry  CHS 4100L – Radiochemical Techniques Lab |
| Indiana University | Chemistry |  | C565 – Nuclear Chemistry |
| Michigan State University | Chemistry |  | CEM 485 – Modern Nuclear Chemistry  CEM 985 – Selected Topics in Nuclear Chemistry |
| Oregon State University | Engineering | Nuclear Engineering | NSE 536 – Advanced Radiation Detection and Measurement |
| Stony Brook University | Chemistry |  | CHE 361 – Nuclear Chemistry  CHE 362 – Nuclear Chemistry Laboratory |
| Texas A&M University | Chemistry |  | CHEM 660 – Nuclear Chemistry  CHEM 661 – Radiochemistry |
| Texas Tech University | Chemistry |  |  |
| University of Alabama, Birmingham | Chemistry |  | CH 477 – Radiochemistry for Life Sciences |
| University of California Berkeley | Chemistry |  | CHEM 143 – Nuclear Chemistry  CHEM 146 – Radiochemical Methods in Nuclear Technology and Forensics  CHEM 243 – Advanced Nuclear Structure and Reactions |
| University of California, Davis | Biomedical Engineering |  |  |
| University of California, Irvine | Chemistry |  | CHEM 133 – Nuclear and Radiochemistry  CHEM133L – Nuclear and Radiochemistry Laboratory |
| University of Central Florida | Chemistry |  |  |
| University of Florida | Nuclear Engineering |  | ENU 4300 – Radiochemistry  ENU 4612 – Nuclear Radiation Detection and Instrumentation |
| *University of Iowa* | *Chemistry* | *Radiochemistry Graduate Certificate* | *CHEM 4760* – *Radiochemistry: Energy, Medicine, and the Environment*  *New Radiochemistry Graduate Certificate Courses* |
| University of Maryland, College Park | Chemistry |  | CHEM 403 – Radiochemistry |
| University of Missouri | Chemistry |  | CHEM 8600 – Radiochemistry and Detection with Lab  CHEM 8610 – Advanced Radiochemistry  CHEM 8630 – Radiopharmaceutical Chemistry |
| University of Nevada Las Vegas | Chemistry | Radiochemistry | CHEM 312 – Introduction to Radiochemistry |
| University of Notre Dame | Civil and Environmental Engineering and Earth Sciences |  | CE 40382 – Actinide Chemistry  CE 60390 – Nuclear Forensic Analysis |
| University of Tennessee | Nuclear Engineering | Radiochemistry Graduate Certificate Program | NE 415 – Introduction to Radiochemistry  NE 416 – Introduction to Radiochemistry Lab  CHEM 580 – Radio and Nuclear Chemistry |
| University of Utah | Chemistry |  | CHEM 3200 – Radiochemistry I |
| Washington State University | Chemistry | Radiochemistry | CHEM 521 – Radiochemistry and Radiotracers  CHEM 522 – Radiochemistry Laboratory  CHEM 550 – Special Topics in Nuclear Processes and Radioactive Waste Management |
| Washington University in St. Louis | Chemistry |  | CHEM 4035/5035 – Nuclear and Radiochemistry Lab |

**Table S2.** Comparison of selected U.S. Radiochemistry Programs that offers a certificate program as a benchmarking summary

| **Program** | **Admission Flexibility** | **Course content (12 semester hour (sh) total for each)** | **Online Delivery** | **Hands-On Training** |
| --- | --- | --- | --- | --- |
| University of Iowa (UI) | Open to enrolled graduate students and working professionals as non-degree seeking students | Modular with eight 1 sh lecture courses taken sequentially and two 2 sh laboratory courses | Yes – asynchronous coursework | Required – two-week intensive lab |
| University of Tennessee (UTK) | Restricted to enrolled graduate students | Two required 3 sh courses (CHEM 580 – Radio and Nuclear Chemistry and NE 550 – Radiation Measurements Laboratory) and two 3 sh electives chosen from NE 433 – Principles of Health Physics, CHEM 511 – Analytical Separations, and CHEM 531 –Materials Inorganic Chemistry and Catalysis | No | Required lab course NE 550 – Radiation Measurements Laboratory |

While there are other programs that offer courses in radiochemistry (**Table S1**), there are no other radiochemistry graduate certificate programs in the U.S. that are designed for continuing education in the workforce. One radiochemistry certificate program is offered at the University of Tennessee and the criterion for acceptance is the same as being admitted into the graduate programs in chemistry or nuclear engineering and all courses are offered in-person with traditional lectures or labs (**Table S2**). The University of Iowa Radiochemistry graduate certificate is available to current graduate students in UI programs and external applicants who apply as non-degree seeking graduate students to enable flexibility for state and federal laboratory workers and industrial partners.

# **S2. PROGRAM AT A GLANCE – UNIVERSITY OF IOWA RADIOCHEMISTRY GRADUATE CERTIFICATE**

**Admission Criteria**

• BS or BA degree with at least two semesters of undergraduate chemistry coursework
• Minimum GPA of > 2.5
• Open to University of Iowa students, APHL fellows, and external professionals through non-degree seeking admission

**Program Structure**

• 12 semester hours total (10 courses) with 8 semester hours online (asynchronous, 1 s.h. each) + 4 semester hours laboratory (two in-person courses, 2 s.h. each, summer block)

• Courses divided into modular components for flexibility in pacing and course load

**Required Online Courses (1 s.h. each)**

• CHEM 5121: Nuclear Physics Concepts
• CHEM 5122: Radiochemistry Separation Concepts I
• CHEM 5123: Radiochemistry Separation Concepts II
• CHEM 5124: Radiochemistry Instrumental Analysis I
• CHEM 5125: Radiochemistry Instrumental Analysis II
• CHEM 5126: Radiochemistry Data Analysis and Statistics
• CHEM 5127: Radiochemistry Quality Assurance
• CHEM 5128: Radiation Safety and Health Physics

**Required Laboratory Courses (2 s.h. each) and Their Competencies**

• CHEM 5129: Radiochemistry Separation Laboratory (2 s.h.) – Covers EPA Methods 900.0, 903.1, 904.0, and 906.0

• CHEM 5130: Radiochemistry Instrumental Analysis Laboratory (2 s.h.) – Gamma Spectrometry, Liquid Scintillation Counting (LSC), Alpha Scintillation, Alpha Spectrometry, and Gas-Flow Proportional Counting (GFPC)

**Pacing Options**

• Ideal completion: 3 semesters (Fall–Spring–Summer) within one year
• Flexible completion: May extend to up to 6 semesters depending on student availability and obligations

**Expected Weekly Commitment**

• Online courses (1 s.h. each): ~4 hours/week per course (asynchronous lectures, assignments, and discussions)
• Laboratory block (2 s.h. each): Intensive ~40 hours/week (laboratory and assignments) for two weeks, full-time in-person

**Program Learning Outcomes (PLO)**

1. Evaluate the chemical and nuclear properties of radionuclides to apply appropriate methodologies for the purification and manipulation of radioactive materials
2. Evaluate the use of instrumentation to perform various radiochemical analyses and apply instrumental analysis technique across a wide range of sample types and radionuclides.
3. Apply radiation safety concepts and evaluate potential risks associated with radiation as they relate to radiochemical manipulation and measurement.
4. Analyze data generated from radiochemical analysis and measurement ensuring complete results and defensible practices.

| **Course Number/Name** | **PLO 1** | **PLO 2** | **PLO 3** | **PLO 4** |
| --- | --- | --- | --- | --- |
| CHEM 5121: Nuclear Physics Concepts | Introduce |  |  |  |
| CHEM 5122: Radiochemistry Separation Concepts I | Introduce |  |  |  |
| CHEM 5123: Radiochemistry Separation Concepts II | Reinforce |  |  |  |
| CHEM 5124: Radiochemistry Instrumental Analysis I |  | Introduce |  |  |
| CHEM 5125: Radiochemistry Instrumental Analysis II |  | Reinforce |  |  |
| CHEM 5126: Radiochemistry Data Analysis and Statistics |  |  |  | Introduce |
| CHEM 5127: Radiochemistry Quality Assurance |  |  |  | Introduce |
| CHEM 5128: Radiation Safety and Health Physics |  |  | Introduce |  |
| CHEM 5129: Radiochemistry Separation Laboratory | Master |  | Master |  |
| CHEM 5130: Radiochemistry Instrumental Analysis Laboratory | Master |  |  | Master |

# **S3. COURSE DESCRIPTIONS**

**CHEM 5121: Nuclear Physics Concepts:** This course introduces the fundamental concepts describing the behavior of unstable nuclei. These concepts include radioactive emissions, radionuclide generation, and interaction with the physical world. It also covers the production and modes of decay of radionuclides, the concepts of radioactive decay and equilibrium, and radiation’s interactions with matter. Additionally, this course discusses the occurrence and importance of natural and anthropogenic radionuclides relative to their use and importance to the environment and human health.

**CHEM 5122/5123: Radiochemistry Separation Concepts I/II:** These courses cover the chemical and physical manipulation samples to enable the identification and measurement of radionuclides. These concepts focus strongly on practical inorganic and analytical chemistry techniques, including sample handling, sample dissolution/destruction, oxidation state manipulation, equilibrium reactions, and analyte preconcentration. Additionally, these courses will address the analytical implications of various sample types, including matrix and radionuclides interferences, and various techniques for addressing these issues.

**CHEM 5124/5125: Radiochemistry Instrumental Analysis I/II:** These courses cover the theory, operation, calibration, and maintenance of instrumentation used for the identification and measurement of radiation. Instrumentation covered by these two courses include gas-flow proportional counters, Geiger-Müller counters, ionization chambers, liquid scintillation counters, semiconductor detectors, and solid scintillation detectors. For each technology introduced, students will learn the fundamental concepts of detection, operation, calibration, troubleshooting, maintenance, and sample measurement.

**CHEM 5126: Radiochemistry Data Analysis and Statistics:** This course introduces the mathematical concepts and calculations used in radiochemistry to calculate analytical results and assess data. These concepts include radioactivity calculations, counting statistics, detection limit decisions, and uncertainty estimations used in the generation of analytical results. This course will also cover the optimization and evaluation of analytical systems to meet data and measurement quality objectives using the previously listed concepts.

**CHEM 5127:** Radiochemistry Quality Assurance: This course covers various components of quality assurance that govern radiochemical analysis. These components include the traceability of measurements, the standardization of measurements systems, process of obtaining and maintaining laboratory accreditation, the various quality control procedures used to ensure defensible data, and the regulatory standards impacting radiochemical measurements. The course will also cover the best practices for method and instrument validation, and the statistical concepts and tests for evaluating radiochemical data.

**CHEM 5128: Radiation Safety and Health Physics:** This course introduces radiation safety and health physics concepts that are used to both minimize human exposure to radiation and evaluate its potential effect. These concepts include processes for minimizing and evaluating exposure, radioactive materials handling and exposure, licensure considerations, and dosimetry. This course will also discuss the biological effects of radiation, including both acute and chronic exposure outcomes, and the regulatory limits meant to minimize these effects.

**CHEM 5129: Radiochemistry Separation Laboratory:** This laboratory course introduces students to the radiochemistry laboratory environment and covers commonly used radiochemical separation techniques. This course covers fundamental laboratory safety and techniques, including exposure and contamination control, waste disposal, sample preparation and preservation, and standard preparation and verification. Students will also perform radiochemical separation methods for gross alpha radium, tritium, and uranium using a variety of techniques including co-precipitation, distillation, extraction chromatography, and ion exchange.

**CHEM 5130: Radiochemistry Instrumental Analysis Laboratory:** This laboratory course introduces students to radiation measurement instruments and data analysis software. This course covers the set-up, calibration, maintenance, and use of alpha scintillation counters, gamma spectrophotometers, gas-flow proportional counters, and liquid scintillation counters. Students will perform setup, calibration, and analysis with each of the listed instruments and their associated software packages.

# **S4. PRACTICAL SKILLS RUBRIC FOR LABORATORY COMPONENTS**

Student performance is assessed via a multi-factor approach including attendance, observation, quizzes, and a summary laboratory report.

| Criteria | Day 1 | Day 2 | Day 3 | Day 4 | Day 5 | Total |
| --- | --- | --- | --- | --- | --- | --- |
| Attendance | 5 pts | 5 pts | 5 pts | 5 pts | 5 pts | 25 pts |
| Completion of Laboratory Activities | 5 pts | 5 pts | 5 pts | 5 pts | 5 pts | 25 pts |
| Daily Quiz | 5 pts | 5 pts | 5 pts | 5 pts | 5 pts | 25 pts |
| Summary Laboratory Report |  |  |  |  |  | 25 pts |
| Total | 15 pts | 15 pts | 15 pts | 15 pts | 15 pts | 75 pts |

**Attendance and Participation:** Attendance, participation, and completion of the various course activities during each week-long course comprise 50% of the students’ grades. Each course activity generates an output document (reviewed and annotated data analysis packet, calculated result worksheet, or sample preparation worksheet) that is reviewed by the course instructor or teaching assistant each day. If students attend each day’s laboratory training session and complete the daily coursework, comprised of practical, hands-on work with radiochemical methods, instrumentation, and data analysis, full credit is awarded for each day. Partial credit is awarded if students are absent and complete the coursework during the week or do not fully participate in each day’s activities.

**Quizzes:** A quiz covering each day’s activities and critical learning outcomes is used to assess student learning and provide reinforcement of key course content. These five daily quizzes comprise 25% of the student’s grade. Each quiz is composed of five multiple-choice or true/false questions. Feedback on each question is provided immediately upon submitting the quiz through the University’s learning management system.

**Summary Laboratory Report:** A written laboratory report covering each week-long course is used to assess student understanding of the coursework and ability to critically assess the materials covered. This summary laboratory report, including an abstract, introduction, materials and methods, discussion, and conclusion covering the week’s laboratory activities, comprises 25% of the student’s grade. The laboratory report is due one week following the end of the in-person course and is assessed for format and quality (20%) and content (80%) based on the rubric included below.

| Criteria | Rating | | | |
| --- | --- | --- | --- | --- |
| Format and Quality | 5 pts: Lab report submitted on time. Minimal errors found. Directions were followed. Few, if any, formatting issues were found. | 3 pts: Minor errors in formatting were found. | 1 pts: Directions were not followed. Major factual or formatting errors were found. Report sections were ambiguous. | 0 pts: No submission/very incomplete. |
| Abstract | 5 pts: Clearly describes the content and purpose laboratory coursework in a succinct manner. | 3 pts: Describes the content or purpose of laboratory course work but is overly long or incomplete. | 1 pts: Poor description of course purpose or content, incomplete. | 0 pts: Section not included/very incomplete. |
| Introduction | 5 pts: Clear background information based on cited methods and literature search. Uses proper citations. Includes a rationale for each laboratory activity. | 3 pts: Contains background information but is not complete. Does not cover all activities in necessary detail. | 1 pts: Very little or no background information. No citations. | 0 pts: Section not included/very incomplete. |
| Materials and Methods | 5 pts: Contains a complete description of each laboratory activity performed and is easy to follow. Includes information on the “why” of the steps for each analysis or activity. | 3 pts: One or more laboratory activity is not included or described completely. Organizational issues present. Understanding of the “why” of the steps for each analysis or activity partially demonstrated. | 1 pts: Multiple laboratory activities are omitted or not described sufficiently. Understanding of the “why” of the steps of each analysis or activity is not demonstrated. | 0 pts: Section not included/very incomplete. |
| Results and Discussion | 5 pts: The key outcomes of each laboratory activity were covered in detail and presented clearly. Report includes tables and figures covering the results of the laboratory activities. Discussion of each laboratory activity demonstrates complete/substantial understanding and introspection on the purpose of the activity. Includes substantial summary information about what the student learned. | 3 pts: One or more laboratory activities were not covered in detail. Report contains no tables or figures covering laboratory activities. Discussion of one or more laboratory activities was incomplete or omitted. Includes some summary information about what the student learned. | 1 pts: Multiple laboratory activities were not covered in detail. Discussion of multiple laboratory activities was incomplete or omitted. Does not convey what the student learned during the course. | 0 pts: Section not included/very incomplete. |

# **S5. PRELIMINARY PROGRAM OUTCOMES**

The University of Iowa Radiochemistry Graduate Certificate Program has graduated its first cohort as of summer 2025, providing an opportunity to capture early outcome data. While still limited, these preliminary results offer insight into the program’s reach, completion trends, and student demographics. **Table S3** summarizes key preliminary program outcomes, including applicant and enrollee counts, completion rates, time-to-completion, fellowship uptake, and demographic information across sector, degree, and geography. Post-certificate outcomes, such as career advancement and workplace impact, are not yet available but will be assessed through planned follow-up surveys in future cohorts. These preliminary findings demonstrate both successful program delivery and opportunities for expanded evaluation.

**Table S3. Preliminary outcome metrics for the first cohort of the University of Iowa Radiochemistry Graduate Certificate Program.**

| **Category** | **Preliminary Outcomes (First Cohort)** |
| --- | --- |
| Applicants | 12 applicants – Open to APHL scholarship recipients only for first cohort |
| Enrollees | 12 enrolled |
| Completion Rate | 100% completed |
| Time-to-Completion | 3 semesters (August 2024 to August 2025) |
| Fellowship Uptake | 100% of students received support from APHL scholarship |
| Demographics – Sector | 100% public health laboratories |
| Demographics – Degrees held | 92% Bachelors, 8% Ph.D. |
| Demographics – Geography | Throughout the United States |
| Post-Certificate Outcomes | Not yet available for first cohort; planned follow-up survey in future cohorts |

**Of note, the program outcomes presented here are operational and aggregated program metrics (non-human subjects). Any future reporting of trainee-level or individually identifiable data will undergo Institutional Review Board (IRB) review as appropriate.*

**Future Work**

Upcoming cohorts will incorporate additional evaluation measures, including pre/post content assessments tied to learning outcomes and 3–6 month follow-up surveys to capture post-certificate impacts such as workplace transfer of skills and career advancement.

# **S6. COSTS AND LOGISTICS**

Costs can be broken down into tuition for the certificate course offerings and travel costs to attend the in-person laboratory courses. At the University of Iowa, tuition costs are based upon admission of students a non-degree student (certificates are not included in the same classification as the MS or Ph.D.) in a hybrid learning center. If students complete the degree in three semesters, the total cost for tuition and fees for the 2025-2026 year is $8703.75. Tuition rates are established by the Iowa Board of Reagents and updated tables for programs in the College of Liberal Arts and Sciences are provided here: <https://tuition.ais.its.uiowa.edu/rates>. The first cohort of students was limited to APHL scholarship recipients that covered tuition and fees for the courses. The second cohort of students include both APHL scholarship recipients and students from other sectors.

Costs to attend the in-person laboratory include flights/transportation, housing costs, and food. These will vary depending on the home location of the student and personal choices associated with the stay. Students are responsible for arranging travel and lodging for the in-person component of the program, although the APHL scholarship program does provide support with travel logistics.

# **S7. STATE HYGIENIC LABORATORY (SHL) CONSTRAINTS AND REGIONAL HUB MODEL CONSIDERATIONS**

The two, one-week intensive laboratory courses are currently held at the State Hygienic Laboratory at the University of Iowa. Currently the laboratory course can accommodate 18 students at the State Hygienic Laboratory during the summer, and this limitation is due to competing work, staffing, and space constraints; future cohorts will also have access to Department of Chemistry Laboratory space for any overflow needs. We expect to be able to handle 30 students per summer at the University of Iowa. Additional capacity could be met by offering laboratory sections during the fall or winter semesters, but that would then delay certificate conferral by an additional four to six months.

To increase capacity and to make completing the certificate easier for more students, we are also proposing a regional hub model. In this framework, we would develop partnerships with training centers in the regions throughout the country to offer the laboratory courses at licensed facilities. This would enable greater capacity for training and improve student offerings due to decreased travel cost and provide more flexibility and course timing. Laboratory sessions held at regional hubs could either be offered and managed by academic institutions in the region or by the University of Iowa. If they were managed by other academic institutions, instructors, course numbers, tuition and fees, and travel logistics would be the responsibility of the regional hub. These courses would be transferable to the University of Iowa for degree completion based on partnerships established between the two institutions. These could be courses already outlined in Table S1 if there were similar learning objectives covered in the laboratory experiments. If they were managed by the University of Iowa, then the curriculum would be established, and the instructor would travel to that site to complete the laboratory course. The partner institution would not need to be an academic institution (providing additional flexibility) but would need a designated radiochemistry laboratory training area and equipment (alpha scintillation counters, gamma spectrometers, gas-flow proportional counters, and liquid scintillation counters). An agreement would need to be in place between the University of Iowa and the hosting institution on responsibilities of each partner. Finally, we want to reiterate that the regional hub model is currently not offered but could be implemented as part of a coordinated national effort to improve radiochemistry workforce training in the United States.
